# Overexpression of Msx1 in Mouse Lung Leads to Loss of Pulmonary Vessels Following Vascular Hypoxic Injury

**DOI:** 10.3390/cells10092306

**Published:** 2021-09-03

**Authors:** James West, Anandharajan Rathinasabapathy, Xinping Chen, Sheila Shay, Shanti Gladson, Megha Talati

**Affiliations:** Division of Allergy, Pulmonary and Critical Care Medicine, School of Medicine, Vanderbilt University, Nashville, TN 37232, USA; j.west@vumc.org (J.W.); anandharajan.rathinasabapathy@vumc.org (A.R.); pchen@geneticsassociates.com (X.C.); sheila.shay@vumc.org (S.S.); santhi.gladson@vumc.org (S.G.)

**Keywords:** pulmonary arterial hypertension, Msx1 expression, mouse models, pulmonary vascular endothelial cells, pulmonary vascular smooth muscle cells, BMP pathway, idiopathic PAH, heritable PAH, RNA sequencing

## Abstract

Pulmonary arterial hypertension (PAH) is a progressive lung disease caused by thickening of the pulmonary arterial wall and luminal obliteration of the small peripheral arteries leading to increase in vascular resistance which elevates pulmonary artery pressure that eventually causes right heart failure and death. We have previously shown that transcription factor Msx1 (mainly expressed during embryogenesis) is strongly upregulated in transformed lymphocytes obtained from PAH patients, especially IPAH. Under pathological conditions, Msx1 overexpression can cause cell dedifferentiation or cell apoptosis. We hypothesized that Msx1 overexpression contributes to loss of small pulmonary vessels in PAH. In IPAH lung, MSX1 protein localization was strikingly increased in muscularized remodeled pulmonary vessels, whereas it was undetectable in control pulmonary arteries. We developed a transgenic mouse model overexpressing MSX1 (MSX1^OE^) by about 4-fold and exposed these mice to normoxic, sugen hypoxic (3 weeks) or hyperoxic (100% 02 for 3 weeks) conditions. Under normoxic conditions, compared to controls, MSX1^OE^ mice demonstrated a 30-fold and 2-fold increase in lung Msx1 mRNA and protein expression, respectively. There was a significant retinal capillary dropout (*p* < 0.01) in MSX1^OE^ mice, which was increased further (*p* < 0.03) with sugen hypoxia. At baseline, the number of pulmonary vessels in MSX1^OE^ mice was similar to controls. In sugen-hypoxia-treated MSX1^OE^ mice, the number of small (0–25 uM) and medium (25–50 uM) size muscularized vessels increased approximately 2-fold (*p* < 0.01) compared to baseline controls; however, they were strikingly lower (*p* < 0.001) in number than in sugen-hypoxia-treated control mice. In MSX1^OE^ mouse lung, 104 genes were upregulated and 67 genes were downregulated compared to controls. Similarly, in PVECs, 156 genes were upregulated and 320 genes were downregulated from siRNA to MSX1^OE^, and in PVSMCs, 65 genes were upregulated and 321 genes were downregulated from siRNA to MSX1^OE^ (with control in the middle). Many of the statistically significant GO groups associated with MSX1 expression in lung, PVECs, and PVSMCs were similar, and were involved in cell cycle, cytoskeletal and macromolecule organization, and programmed cell death. Overexpression of MSX1 suppresses many cell-cycle-related genes in PVSMCs but induces them in PVECs. In conclusion, overexpression of Msx1 leads to loss of pulmonary vessels, which is exacerbated by sugen hypoxia, and functional consequences of Msx1 overexpression are cell-dependent.

## 1. Introduction

Pulmonary arterial hypertension (PAH) is a rare cardiopulmonary disease [1,2,3,4,5]. The prominent pathologic features of PAH are irreversible loss of small peripheral pulmonary arteries with widespread obliterative vasculopathy, resulting in progressive elevation of pulmonary arterial pressure that leads to right ventricular failure and death. In PAH, disorganized growth of pulmonary artery cells, impaired vascular regeneration, and ectopic blood vessel formation that leads to plexiform lesions are believed to prevent blood flow to the capillaries and cause progressive loss of small pulmonary arteries [6,7]. Despite increasing understanding of the genetic and molecular mechanism behind PAH, the field of disease progression is still unclear. Therefore, currently available therapeutic strategies are not successful in targeting the persistent loss of small pulmonary arteries and vascular remodeling [7] and warrant studies to understand disease progression in PAH.

Group 1 form of PAH is divided mainly into two forms: (1) heritable form of the disease (HPAH) which is largely associated with mutation of BMPR2; and (2) idiopathic form of the disease (IPAH) [8]. Clinical features of disease in HPAH and IPAH are largely indistinguishable, although patients with HPAH have an earlier age of onset with worse hemodynamic features upon initial diagnosis [9]. Similarly, while a few moderate risk factors for IPAH have been found through candidate gene approaches [10,11,12], major genetic risk factors are still unknown. In patient-derived IPAH and HPAH transformed lymphocyte population, by affymetrix arrays, we show that HPAH and IPAH share the majority of altered signaling pathways. Interestingly, however, while IPAH cases have a shared molecular origin, which is closely related, it is distinct from HPAH [13]. One such strong candidate gene we identified was transcription factor Msx1 (muscle segment homeodomain-like homeobox 1), which is the most upregulated gene (4X) in IPAH patient-derived transformed lymphocytes [13].

Msx1 gene encodes a homeodomain-containing protein that functions as a transcriptional repressor [14,15]. It is expressed at sites where cellular proliferation and apoptosis occur during pattern formation during embryogenesis [16], such as formation of the neural tube, the limb buds, and derivatives of the cranial neural crest [17,18]. Classically, Msx1 gene expression maintains cells in a pluripotent state [19], and a tight link between Msx1 sense and anti-sense RNAs regulates the expression of Msx1 required for adult homeostasis [19,20]. Msx1 is a downstream effector of the bone morphogenic protein (BMP) pathway and is therefore regulated by BMP pathway [21]. For example, BMP signaling pathway is shown to regulate Msx1 expression during odontogenesis [22]. Similarly, in newborn rat pupillary membrane, during development, activation of BMP pathway leads to upregulation of Msx1. This is shown to contribute to endothelial apoptosis in a paracrine fashion resulting in programmed capillary regression [23]. BMP signaling through SMADs is required for transcriptional activation of the Msx1 gene [24].

Under pathological conditions, genes active during embryogenesis can become reactivated [25]. It is seen that Msx1 when activated under such circumstances can prevent terminal differentiation of progenitor cells and can dedifferentiate terminal differentiated cells [26,27]. In collateral arteries, fluid sheer stress can also highly induce Msx1 expression, leading to inflammation-driven arteriogenic remodeling [21]. In cultured cells, overexpression of Msx1 inhibits cell proliferation and induces cell apoptosis [28,29,30]. Dysregulation of BMP pathway can contribute to reactivation of Msx1 transcription [31]. In vascular smooth muscle cells, BMPs, via induction of Msx, trigger downregulation of SMC marker gene expression [32]. In PAH (both IPAH and HPAH), dysregulation of BMP pathway has been well established [33]. Using Affymetrix assays, we have demonstrated significant increase in expression of Msx1 gene in IPAH and HPAH patient-derived transformed lymphocytes [13].We therefore sought to investigate the role of increased Msx1 expression in PAH, specifically in pulmonary arteries. 

## 2. Methods

### 2.1. Animal Model

MSX1-overexpressing mice were created by driving the canonical mouse MSX1 sequence (912 bp/303 amino acids) in a single exon with a TetO_7_-CMV promoter, with transcriptional start indicated by a canonical Kozak consensus sequence and terminated by the SV40 polyadenylation sequence and intron. Pronuclear injection was used to introduce this construct into mice. TetO7-CMV Msx1 mice were crossed to Rosa26-rtTA2 mice [34] (Rosa26 or control mice) to create mice with universal overexpression of Msx1 only when induced by doxycycline. Rosa26-only mice were used as controls.

**Figure 1:**
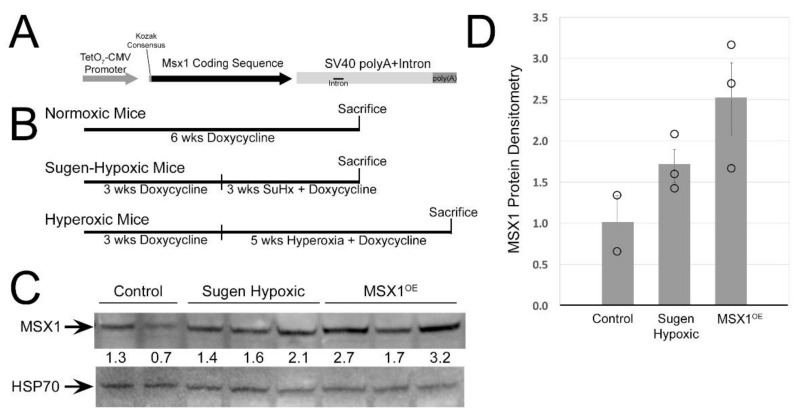
MSX1 expression is increased in Msx1^OE^ mice. Msx1 (**A**) design of the construct used to make the MSX1^OE^ mice; they were then crossed with Rosa26-rtTA2 mice, and fed doxycycline to initiate expression. (**B**) Schematic representation of experimental design for each treatment. (**C**,**D**) Protein expression is increased in Msx1^OE^ mice compared to control and sugen-hypoxia-treated mice.

Studies were conducted in mice (aged 25–33 weeks at sacrifice) given 200 mg/kg doxycycline for six weeks to activate the transgene. The mice (Figure 1B) were either: (1) kept in normoxic conditions for 6 weeks; or (2) treated with a single subcutaneous injection of 20 mg/kg of Sugen 5416 (Sigma, St. Louis, MO, USA) in dimethyl sulfoxide (Sigma, St. Louis, MO, USA) after 3 weeks of dox treatment and then placed in 10% hypoxia for three more weeks [35]; or (3) kept in a hyperoxic chamber with 95% of O_2_ for 3 h daily for 5 weeks after 3 weeks of dox treatment [36]. Hyperoxia-treated mice were positive controls for vessel loss in the retina [37]. All the animals underwent echocardiography at the time of sacrifice and invasive hemodynamics was performed through a closed-chested technique for measurement of right ventricular systolic pressure (RVSP). Blood was drawn for blood glucose measurements and assayed by the Mouse Metabolic Phenotyping Core at Vanderbilt University Medical Center. All animal procedures were approved by the Vanderbilt Institutional Animal Care and Use Committee (IACUC protocol M-12-090).

### 2.2. Immunostaining for Human Paraffin-Embedded Sections

Immunolocalization of MSX1 was performed on archival paraffin-embedded human lung tissue obtained from controls and IPAH patients. The study protocol was approved by the Institutional Review Board of Vanderbilt University Medical Center (IRB protocol 111066). Lung sections were deparaffinized, rehydrated, and blocked with 5% normal goat serum, followed by overnight incubation with primary antibody (MSX1, ab49153, Abcam) at 4 °C. The sections were then incubated with biotinylated secondary antibody followed by incubation with HRP-conjugated streptavidin. Diaminobenzidine (DAB) was used as a substrate for HRP. The sections were dehydrated and mounted in Cytoseal XYL (Richard-Allan Scientific, Kalamazoo, MI, USA) for light microscopic examination.

### 2.3. Measurement for Pulmonary Vessels

After euthanizing the mice, the left atrium was removed, and the pulmonary circulation was perfused with PBS using a syringe-generated flow. The left lung was inflated with 0.8% low melt agarose at constant inflation pressure and embedded in paraffin. Samples were transversely sectioned at 6 µm thickness and immune-stained with antibody against smooth muscle alpha actin (αSMA, 4 °C overnight, M0851 DAKO) followed by 1 h (at RT) incubation with fluorescent-tagged (goat anti rabbit 594, A32740 Invitrogen) secondary and counterstained with DAPI. Random fields were selected with DAPI, avoiding fields with large airways or bronchi, and then vessel size and muscularization were measured for each vessel identified by αSMA staining. A total of 30–50 arteries at 100× magnification were selected per mouse lung for analysis.

### 2.4. Isolectin Staining of Mouse Retina

First of all, mouse retina was dissected for whole mount [38]. For isolectin staining [39], briefly, −20 °C methanol-fixed retinas were washed in PBS and then covered with 100 μL of Perm/Block solution (PBS + 0.3% Triton + 0.2% BSA) for 1 h with gentle shaking. This was followed by gentle shaking overnight at 4 °C with IsolectinB4-Alexa488 (I21411, Thermo Fisher Scientific/Life Technologies, Waltham, MA, USA). The next day, retinas were washed in PBS + 0.3% Triton and mounted by adding 50 μL of Prolong mountant onto a coverslip and gently positioning over the retina. Slides were kept in a refrigerator overnight at 4 °C, ensuring that they were flat and protected from light, to allow the Prolong mountant to set and be imaged using a confocal microscope. The mask of the red channel was created, and the total area of the network was acquired by ImageJ measurement. Measurements of all petals of dissected retina were collected to calculate the vascular percentage of the whole retinas.

### 2.5. Western Blotting of Mouse Lung

Mouse lung tissue was homogenized in RIPA buffer (PBS, 1% Ipegal, 0.5% sodium deoxycholate, 0.1% SDS) with proteinase and phosphatase inhibitor cocktails (Sigma-Aldrich). Protein concentration was determined by Bradford assay (Pierce, CO, USA) and was stored at −70 °C until use. Primary antibodies used for Western blot included MSX1 (ab49153, Abcam) and HSC70/HSP73, referred to as HSP70 (ADI-SPA-816, Enzo). Donkey anti-rabbit (711-035-152, Jackson ImmunoResearch Laboratories, Inc., West Grove, PA, USA) was used as secondary antibody.

### 2.6. RNA Sequencing of Mouse Lung and Cultured Mouse Cells

RNA isolated from the 3 control and 3 MSX1^OE^ mouse lung and from PVECs and PVSMCs transfected with Msx1 plasmid or Msx1 siRNA was submitted for RNA sequencing to Vanderbilt Technologies for Advanced Genomics Core at Vanderbilt University Medical Center. All samples with RNA integrity number RIN values greater than 6 were submitted for sequencing analysis. RNA sequencing was performed on an Illumina HiSeq system with a paired-end mRNA library prep, PE-150, with 30 million reads. Initial alignment and quantification of sequences was performed using the Partek Flow package. STAR 2.5.3a was used to align RNA-Seq reads, with quantification to Ensembl Transcripts Release 83 using Partek E/M. Reads were normalized to total count. Approximately 77% of all reads aligned to genes. A total of 22,232 genes were identified with at least one read in each sample. Genes with fewer than four reads per million were filtered out. Genes were filtered for those that had a statistically significant (*p* < 0.05), >1.4-fold change difference between control and Msx1^OE^ mice. Overrepresentation analysis was completed using WebGestalt (webgestalt.org, accessed on 6 March 2018) for the Mus musculus organism and gene ontology database, comparing against the genome-protein coding database. Multiple test adjustment was conducted using the Benjamini–Hochberg procedure and a false discovery rate threshold of 0.05 or lower was considered significant.

### 2.7. Msx1 Plasmid and siRNA Transfections in Cultured Pulmonary Vascular Endothelial and Pulmonary Vascular Smooth Muscle Cells

Pulmonary vascular endothelial cells (PVECs) and pulmonary vascular smooth muscle (PVSMC) were isolated from conditionally immortalized murine lines generated on the ImmortoMouse [40,41]. Cells were reverted to a primary endothelial or smooth muscle phenotype by removal of murine interferon-gamma and transition to 37 °C (from 33 °C, which maintains conditional immortal phenotype through expression of SV40 large T), for at least 72 h prior to experiments. Msx1 plasmid (MC208982, NM_010835, mouse untagged clone, Origene, Rockville, MD, USA) and Msx1 siRNA (ID17701, Origene) were used for transient transfections of PVECs and PVSMCs in a 6-well plate (in duplicates) with seeding density of 4 × 10^5^ cells per well. Following 48 h of transfections, RNA was isolated from these cells for RNA sequencing.

### 2.8. Statistical Analysis

**Figure 2:**
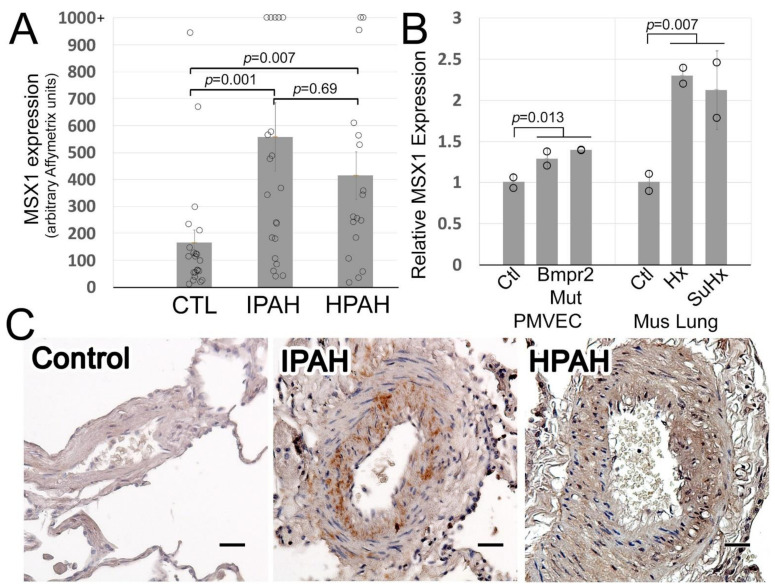
(**A**) MSX1 expression in lymphocytes cultured from control, IPAH, and HPAH patients; each circle is data from one patient, with averages and standard error of the mean indicated by grey bars with error bars. Significance shown was by ANOVA with post hoc tests on log-transformed data (required to satisfy normality). Significant differences were also *p* < 0.01 by Wilcoxon non-parametric test. (**B**) MSX1 is increased both by BMPR2 mutation in isolated pulmonary microvascular endothelial cells (**left**) and by either hypoxia or sugen-plus-hypoxia (**right**) in whole mouse lung. Mice were two sets of pooled RNA from both male and female mice (each circle represents 4 mice/pool). (**C**) MSX1 protein expression is increased in IPAH and HPAH pulmonary arteries. Intense brown color staining in pulmonary vessel cells as indicated by arrows in IPAH and HPAH patients (*n* = 3). Magnification is 200×. Scale bar = 50 μM.

Statistical analyses for continuous variables were carried out using unpaired two-tailed *t* tests and two-way Anova (GraphPad Prism Software, La Jolla, CA, USA). Data were expressed as mean ± SEM. *p* < 0.05 was considered significant. Values were checked for normality by Shapiro–Wilk before running ANOVA with post hoc tests. Data in Figure 2A were not normal in raw form, but were normal when log-transformed. ANOVA on log-transformed and non-parametric tests both found significance.

## 3. Results

### 3.1. Msx1 Protein Expression Is Strikingly Increased in IPAH Pulmonary Arteries

In IPAH transformed lymphocytes, we have shown that MSX1 gene expression is significantly upregulated compared to controls [13]. Individual data from that study show that although there are large individual differences, on average both HPAH and IPAH patients have roughly three times the expression of MSX1 of controls (Figure 2A). Examining MSX1 in BMPR2 mutant pulmonary microvascular endothelial cells [13], there is a slight increase (30–40%) with either a kinase domain truncation or a tail domain truncation BMPR2 mutation (Figure 2B). Moreover, it is upregulated in the whole mouse lung from either hypoxic or sugen hypoxic mice (Figure 2B). We then immunolocalized expression of MSX1 protein in paraffin-embedded lung tissue from IPAH and controls. As shown in Figure 2C, MSX1 protein expression is mainly localized to pulmonary arteries. In control lung, MSX1 protein expression is very low (pale brown color staining in a few cells) in pulmonary vessels; however, in IPAH and HPAH lungs, muscularized pulmonary artery cells show a very strong to moderate positive brown staining, indicating significantly higher expression of MSX1 in these remodeled pulmonary vessels. These data suggest that MSX1 upregulation is a consequence, both directly of Bmpr2 mutation and perhaps indirectly of increased pulmonary vascular pressures.

### 3.2. MSX1 Expression Is Increased in Msx1OE Mice

To understand the role of MSX1 in PAH lung, we first created an MSX1^OE^ mouse model by pronuclear injection with the construct described in Figure 1A. These Rosa26-rtTA2 × TetO_7_-Msx1 mice (referred to hereafter as MSX1^OE^ mice) had a roughly 3.7× increase in MSX1 expression when induced (not shown), which resulted in a roughly 2.6× increase in protein levels (Figure 1C,D) in whole lung. Sugen hypoxic mice, which had a roughly 2× increase in Msx1 expression (Figure 2B) had a roughly 1.5× increase in protein levels.

### 3.3. Hemodynamic Parameters in MSX1^OE^ Mice Following Sugen Hypoxia or Hyperoxia Treatment

**Figure 3:**
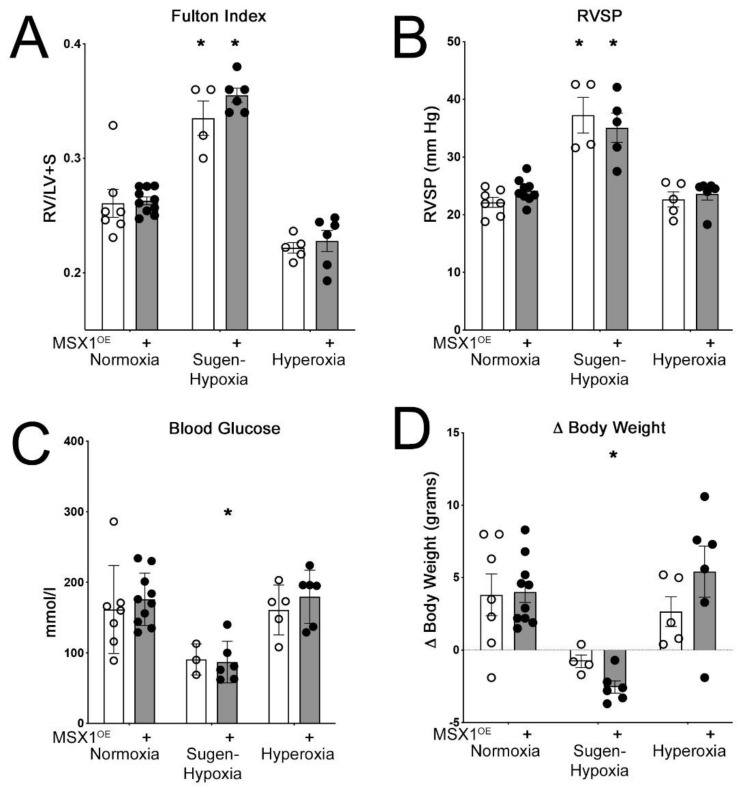
Hemodynamic parameters. (**A**,**B**) Fulton index and RVSP increases following sugen hypoxia treatment in control and MSX1^OE^ mice. (**C**) Blood glucose levels decrease in MSX1^OE^ mice following sugen hypoxia treatment compared to mice under normoxic conditions. (**D**) Body weight decreases in MSX1^OE^ mice following sugen hypoxia treatment compared to mice under normoxic conditions. (* *p* < 0.05—Comparing control and Msx1^OE^ mice from normoxia group with sugen-hypoxia group, respectively). (Open circles control mice and black circles MSX1^OE^ mice.)

In normoxic and hyperoxic group, Fulton index, RVSP, blood glucose, and change in body weight were similar in control and MSX1^OE^ mice (Figure 3A–D). In sugen hypoxia group, as expected [42], Fulton index and RVSP were significantly (*p* < 0.01) increased in both control and MSX1^OE^ mice (but were not different between the two groups) compared to those mice in normoxia group (Figure 3A,B). Further, blood glucose and change in body weight were significantly decreased (*p* < 0.05) (Figure 3C,D) in MSX1^OE^ mice and trended lower in control mice compared to control mice in normoxic group. Interestingly, in sugen hypoxia group, decrease in body weight in MSX1^OE^ mice was significant (*p* < 0.05) compared to control mice (Figure 3D) in this group.

### 3.4. MSX1 Overexpression Leads to Loss of Capillaries in Mouse Retina and Following Sugen Hypoxia Treatment Resulted in Loss of Pulmonary Vessels in MSX1^OE^ Mice

**Figure 4:**
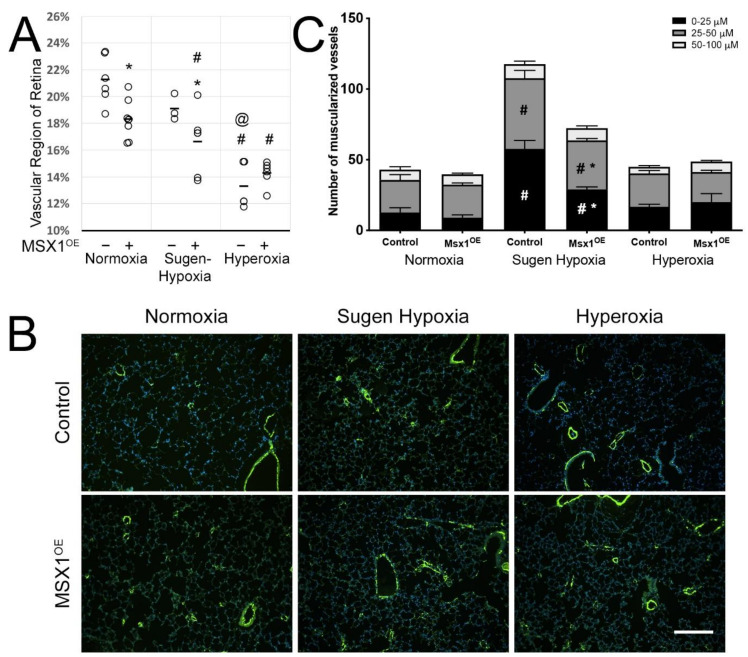
(**A**) Vascular region in the retina is decreased in MSX1^OE^ mice under normoxic and sugen hypoxia conditions compared to controls. (* *p* < 0.01—Comparing Control and Msx1^OE^ mice. # *p* < 0.03—Comparing Msx1^OE^ normoxia group mice with Sugen Hypoxia or hyperoxia group mice. @ *p* < 0.002—Comparing sugen hypoxia group mice with hyperoxia group mice) (**B**) Immunostaining for alpha SMA (pulmonary vessel in green color). Magnification 100×. Scale bar = 50 μM. (**C**) In MSX1^OE^ mice, pulmonary vessels (0–25 μM and 25–50 μM in size) were decreased compared to controls following sugen hypoxia treatment (*n* = 3). (# *p* < 0.02—Comparing control and Msx1^OE^ mice from normoxia group with sugen-hypoxia group, respectively. * *p* < 0.05—Comparing control and MSX1^OE^ mice in sugen-hypoxia group).

Msx1 gene, which is mainly activated during embryogenesis, is shown to be reactivated in adults under pathological conditions [25]. During development, MSX1 upregulation is shown to contribute to endothelial apoptosis in a paracrine fashion resulting in programmed capillary regression [23]. We, therefore, sought to determine whether MSX1 overexpression in MSX1^OE^ mouse leads to capillary regression under normoxic and sugen hypoxic conditions compared to mouse treated with hyperoxia [43]. As shown in Figure 4A, under normoxic conditions, there was modest but significant (* *p* < 0.01) decrease in vascular region in MSX1^OE^ mouse retina (18%) compared to control mouse retina. Sugen hypoxia treatment led to a further decrease in MSX1^OE^ mouse retina (16%, compared to normoxia MSX1^OE^ mice (18%)) as well as in control mice retina (19%, compared to normoxia control mice (21%)); however, it reached statistical significance (# *p* < 0.03) only in MSX1^OE^ mice. In addition, in sugen hypoxia group, decrease in vascular region (16%) in MSX1^OE^ mouse retina was also significant (* *p* < 0.04) compared to control mouse retina (19%) in this group. Under hyperoxic conditions, as expected [36], the vascular region of retina was significantly decreased (# *p* < 0.001) in both control (13%) and MSX1^OE^ (14%) mice compared to the respective groups under normoxic conditions. Further, there was no difference in the percentage of vascular region in MSX1^OE^ mice compared to control mice. When the vascular region of retina from sugen-hypoxia-treated mice was compared with hyperoxic mice, the vascular region of retina was significantly (@ *p* < 0.002) decreased in hyperoxic control mice but not in hyperoxic MSX1^OE^ mice. This suggests that decrease in vascular region in MSX1^OE^ mouse retina following sugen hypoxia treatment is similar to hyperoxia treatment. Taken together, these results indicate that under normoxic conditions, compared to controls, MSX1^OE^ mice demonstrated a significant retinal capillary dropout, which is increased further with sugen hypoxia and is comparable to hyperoxia-induced capillary dropout.

We next investigated whether MSX1overexpression influences pulmonary vessel number in the mouse lung in MSX1^OE^ mice. Under normoxic conditions (Figure 4B,C), in MSX1^OE^ mice, the total number of pulmonary vessels was not significantly different than in controls; however, the number of small pulmonary vessels (0–25 μM), although not significant, appeared to be lower in MSX1^OE^ mice compared to controls. Sugen hypoxia treatment led to approximately 3-fold and 2-fold increase in the total number of muscularized vessels in control and MSX1^OE^ mice, respectively, compared to these mice in normoxia group (Figure 4B,C). In sugen-hypoxia-treated control and MSX1^OE^ mice, the increase in the number of pulmonary vessels was mainly in small (0–25 μm) and medium size (25–50 μM) pulmonary vessels, whereas the number of large size (50–100 μM) pulmonary vessels remained unchanged. In control mice, the number of small pulmonary vessels increased more than 4-fold (# *p* < 0.003) and medium size pulmonary vessels increased 2-fold (# *p* < 0.02) (Figure 4B,C). Similarly, in MSX1^OE^ mice, the number of small pulmonary vessels increased more than 3-fold (# *p* < 0.002) and medium size pulmonary vessels increased 1.5-fold (# *p* < 0.004) (Figure 4B,C). Interestingly, following sugen hypoxia treatment, there was significant decrease in the number of small (50%, * *p* < 0.01) and medium (30%, * *p* = 0.05) size pulmonary vessels in MSX1^OE^ mice compared to control mice (Figure 4B,C). Finally, in hyperoxia-treated control and MSX1^OE^ mice, there was no change in the total number of pulmonary vessels compared to normoxia-treated mice (Figure 4B,C). Taken together, these results indicate that, under sugen hypoxic conditions, compared to controls, MSX1^OE^ mice demonstrated a significant loss of small and medium size pulmonary vessels.

### 3.5. Gene Expression Differences in MSX1^OE^ Mouse Lung Compared to Controls

**Figure 5:**
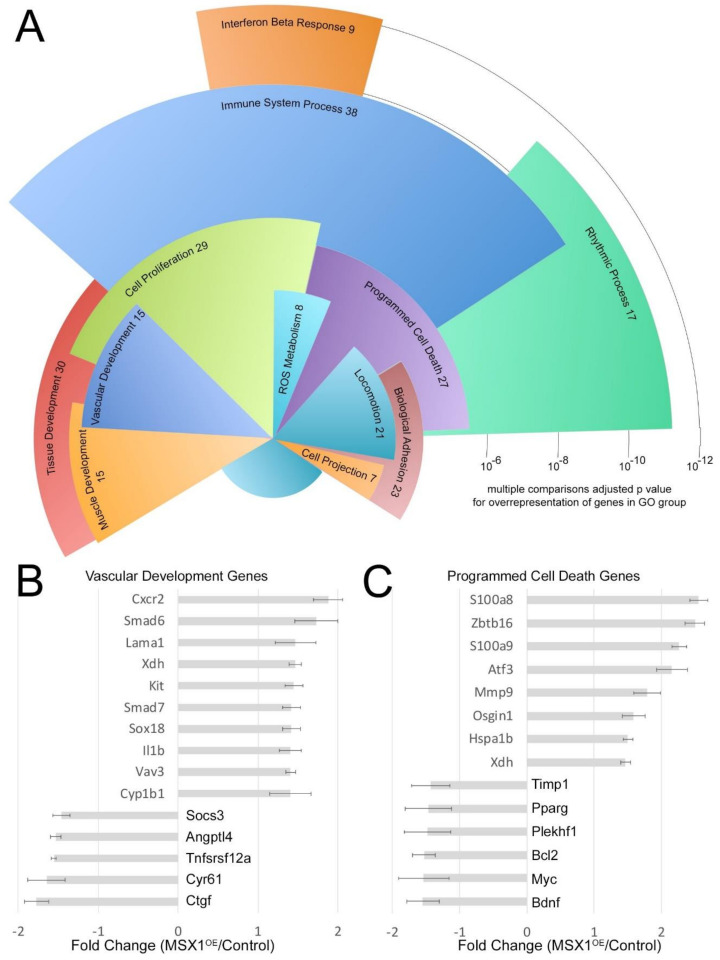
(**A**) Expression arrays comparing Msx1OE mouse lung with controls. (**A**) Overrepresented gene ontology groups from 171 genes with 2-fold changed expression (up or down) in the MSX1^OE^ mouse compared to controls. Radius corresponds to statistical significance; angular width corresponds to number of genes included. (**B**) Relative expression of genes in the vascular development group. (**C**) Relative expression of genes in the autophagy group.

We next sought to determine genes and pathways that are differentially regulated in MSX1^OE^ mouse whole lung compared to controls by RNA sequencing. In the MSX1^OE^ mouse lung, 104 genes were upregulated at least 1.4-fold and 67 genes were downregulated at least 1.5-fold compared to controls, which is a relatively subtle effect. In examination of overrepresented gene ontology groups, 130 of these 171 have GO information, of which 88 fit into overrepresented groups. As shown in Figure 5A, the most significantly overrepresented GO groups include interferon beta response and immune system process, although there was no striking inflammation in the lungs and there were no differences in CBC (data not shown). Of more obvious relevance to PAH were groups such as cell proliferation, ROS metabolism, biological adhesion, tissue development, vascular development, and programmed cell death.

Looking more closely at some of these, the genes that fall within the vascular development gene ontology group contain a few prototypical vasculogenesis genes (Figure 5B) such as an angiopoietin (Angptl4) and the developmental gene Socs3 (both downregulated). However, Smad6 and Smad7 were both upregulated—inhibitors of the BMP and TGFb pathways, respectively. In addition, CYP1B1, which we have previously seen associated with PAH in patients [44], was upregulated by MSX1 overexpression. Similarly, the programmed cell death group (Figure 5C) contains a few prototypical apoptosis genes, such as Bcl2 and Myc, but the majority of genes are multifunctional, with apoptosis being only a minor part of the role they can play. Loss of PPARG, for instance, is strongly associated with disease progression in PAH [45].

### 3.6. MSX1 Gene Expression in PVECs and PVSMCs Are Involved in Cell Cycle, Cytoskeletal and Macromolecule Organization, and Programmed Cell Death

**Figure 6:**
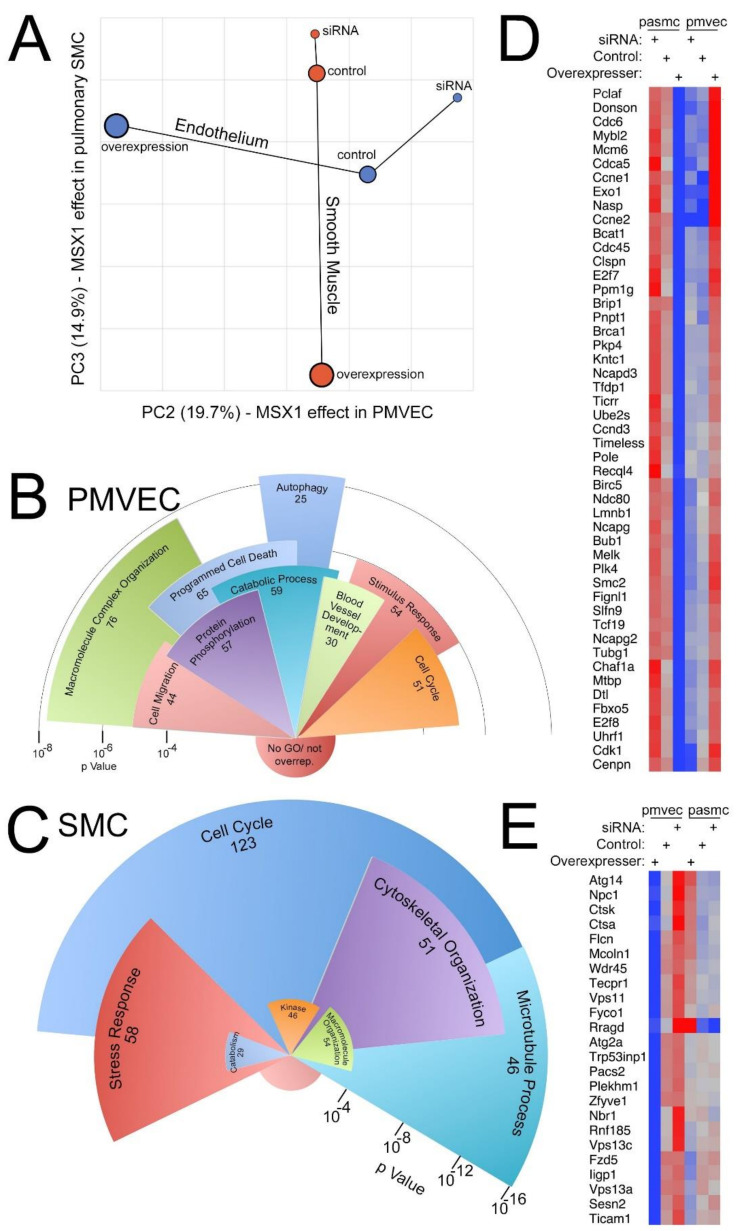
Expression arrays comparing MSX1^OE^ mouse lung with controls. (**A**) Orthogonal effects of MSX1 gene expression in PVECs and PVSMCs. (**B**) Overrepresented gene ontology groups associated with MSX1 gene expression in PVECs. (**C**) Overrepresented gene ontology groups associated with MSX1 gene expression in PVSMCs. (**D**,**E**) Overexpression of MSX1 suppresses many cell-cycle-related genes in PVSMCs but induces them in PVECs. Radius corresponds to statistical significance; angular width corresponds to number of genes included. Red-Gene expression is increased, Blue-Gene expression is decreased.

As gene expression analysis in the whole mouse lung by RNA-seq included GO groups with gene clusters involved in tissue development, cell proliferation, programmed cell death, muscle development, and vascular development, we sought to study the effect of MSX1 gene expression (either overexpression (OE) or silencing MSX1 gene (siRNA)) at the cellular level using mouse PVECs and PVSMCs. As shown in Figure 6A, principle component analysis (PCA) demonstrates, first, that in both endothelium and smooth muscle, siRNA and overexpression pushed the same genes in opposite directions, which is comforting, and second, that the changes in smooth muscle and the changes in endothelium were largely orthogonal—the fact that they are on different principle components suggests that they are not related at all. Principle component 1 is not plotted, because it was simply separation of PMVEC from PASMC (making up 43.5% of total difference). Thus, the first three principle components—the “smooth muscle is not endothelium” component and the two plotted, together make up nearly 80% of total variance.

In PMVECs, 156 genes were upregulated 1.5-fold from siRNA to overexpression and 320 genes were downregulated 1.5-fold from siRNA to overexpression (with controls in the middle). In PVSMCs, 65 genes were upregulated 1.5-fold from siRNA to overexpression and 321 genes were downregulated 1.5-fold from siRNA to overexpression (with control in the middle). As shown in Figure 6B, in PVECs, significantly overrepresented GO groups included, but were not limited to, autophagy, macromolecule complex organization, programmed cell death, blood vessel development, cell migration, and cell cycle. Similarly in PVSMCs (Figure 6C), significantly overrepresented GO groups included, but were not limited to, cell cycle, microtubule process, cytoskeletal organization, and stress response. Interestingly, overexpression of MSX1 suppresses many cell-cycle-related genes in PVSMCs but induces them in PVECs (Figure 6D). By contrast, autophagy genes (Figure 6E), the most statistically significantly overrepresented GO group, were almost universally downregulated by MSX1 overexpression in endothelial cells but were largely unchanged in smooth muscle cells.

## 4. Discussion

In this study, we first demonstrate that in PAH and in mouse models of PH, MSX1 gene expression is augmented and MSX1 protein is prominently localized in remodeled PAH pulmonary arteries. Our newly constructed doxycycline inducible MSX1^OE^ mouse model universally overexpressing MSX1 gene enabled us to gain unique insights into the effects of MSX1 gene and protein overexpression on the pulmonary vasculature, especially following pulmonary vascular injury. Our results indicate that: (1) MSX1 gene overexpression or vascular hypoxic injury leads to increase in MSX1 protein. (2) MSX1 overexpression reduces body weight following sugen hypoxic injury. (3) MSX1^OE^ mice demonstrate a significant retinal capillary dropout, compared to controls, under normoxic conditions, which was further exacerbated with vascular hypoxic injury. (4) MSX1^OE^ mice also demonstrate a significant loss of pulmonary vessels less than 50 uM compared to controls, following vascular hypoxic injury. (5) MSX1^OE^ mouse lung shows significant overrepresentation of GO groups involved in vascular changes such as cell proliferation and programmed cell death that could be prompt loss of pulmonary vessels. (6) Finally, overexpression of MSX1 or blunting MSX1 gene expression in PVECs and PVSMCs indicate an orthogonal effect of MSX1 expression in these cells. Taken together, our data demonstrate that MSX1 overexpression in mouse lung leads to significant pulmonary vessel loss and influences multiple pathway genes associated with vascular development, in a cell-specific manner.

In pathological conditions, MSX1 gene upregulation can prevent terminal differentiation of progenitor cells and induce dedifferentiation of terminal differentiated cells [26,27]. In addition, transient expression of MSX1 in multipotent muscle satellite cells has been shown to retain these cells in a primitive state [46]. In IPAH lung, based on the MSX1 protein localization in remodeled pulmonary arteries, we believe that MSX1 could potentially be localized in the dedifferentiated PASMCs. This is because, according to the current concept of PH pathogenesis, PASMCs can undergo a phenotypic switch from a contractile (differentiated) to a synthetic/proliferative (dedifferentiated) state that drives medial thickening and vascular remodeling in PH [47,48]. It is therefore possible that, in PAH, ectopic MSX1 expression could prompt dedifferentiation of PASMCs and influence remodeling of pulmonary arteries.

In MSX1^OE^ mice, augmented MSX1 protein expression as a result of MSX1 gene overexpression is shown to be accompanied by a modest but significant decrease in the vascular region of retina and a trend towards loss in small pulmonary vessels but no detectable change in Fulton index (function of RVSP). This suggests that in MSX1^OE^ mice, MSX1 gene overexpression may drive pulmonary vascular changes which may be occurring prior to increase in RVSP. We, therefore, believe that this model can potentially be useful to understand the influence of MSX1 overexpression on pulmonary vasculature that can cause or lead to increase in pulmonary pressure, and thus help understand the early events that occur in PAH prior to detectable clinical features, such as increase in RVSP.

Sugen hypoxic injury mouse model, a well-studied model of PH, is shown to develop profound and sustained pulmonary hypertension phenotype with characteristic neointimal smooth muscle cell proliferation and obliteration leading to increased RV pressure and RV dysfunction [42]. In control mice, we observed the expected hemodynamic and pulmonary vascular changes following sugen hypoxic treatment. To our surprise, in MSX1^OE^ mice, there is significant decrease in the number of pulmonary vessels less than 50 uM and retinal vessel loss was accelerated following sugen hypoxia treatment. This suggest that overexpression of MSX1 in mice, can cause damage to pulmonary and retinal cells, and thus inhibits normal intact vessel formation, leading to vessel loss in lung and retina, especially after vascular hypoxic injury. It has been shown that forced expression of Msx1 in mouse muscle tissue inhibits vessel sprouting, and application of an Ad-MSX1-transfected conditioned medium onto the chicken chorioallantoic membrane (CAM) leads to a significant inhibition of new vessel formation [29]. Furthermore, this is thought to occur because MSX1 expression can induce G1/S cell cycle arrest leading to apoptosis [49]. We, therefore, believe that Msx1 overexpression in mouse disrupts the delicate balance required for proper vessel development [50], leading to cell apoptosis of pulmonary and retinal vessels.

Our RNA sequencing data indicate that MSX1 overexpression in either mouse lung, PVECs, or PVSMCs significantly overrepresents various GO groups of relevance to PAH. These include (but are not limited to) cell proliferation, ROS metabolism, biological adhesion, tissue development, vascular development, programmed cell death, autophagy, blood vessel development, cell migration, cell cycle, microtubule process, cytoskeletal organization, and stress response. During embryogenesis, Msx1 is shown to orchestrate gene expression and regulation of many of these pathways, which include cell growth, proliferation, differentiation, cell-to-cell communication, and the apoptotic pathway during pattern formation [32]. Under pathological conditions, genes active during embryogenesis can become reactivated [25]. We believe that overexpression of Msx1 in adult mouse can potentially reactivate many of these pathways. It is also known that dysregulation of BMP pathway can contribute to reactivation of Msx1 transcription [31], as MSX1 is a downstream effector of the BMP pathway [21].

Next, we observed that the changes in PVSMCs and the changes in PVECs are largely orthogonal, suggesting that these cells are not related and function differently because of ectopic Msx1 expression. For example, in our study, overexpression of MSX1 suppresses many cell-cycle-related genes in PVSMCs but induces them in PVECs. Similarly, autophagy genes are almost universally downregulated by MSX1 overexpression in endothelial cells, but are largely unchanged in smooth muscle cells. It has been previously shown in a mouse model of peripheral arterial disease, that during arterial remodeling, functional role of MSX1 and its expression linked to BMP signaling in SMCs is different from ECs [21]. Further, in vitro studies show that altering MSX1 expression affects leukocyte adhesion and ICAM1 expression in ECs but not in SMCs [21]. Similarly, overexpression of Msx1 in HUVECs is shown to inhibit angiogenesis, and silencing of Msx1 by siRNA abrogates its anti-angiogenic effects [29].

Msx1 is known to be a downstream target of the canonical BMP signaling pathway. BMP4 pathway is shown to play pivotal roles in programmed capillary regression in a paracrine manner [23] and mediate apoptotic cell death in developing chick eye [51]. In collateral endothelial cells during arteriogenic remodeling, activation of BMP4 leads to upregulation of MSX genes which correlates with apoptosis [21] and translocation of Smad1 into the nucleus, which appears to be essential for MSX1 induction [21]. In addition, in differentiated cultured VSMCs, BMP-dependent activation of MSX (Msx1 and Msx2) downregulates SMC marker expression by markedly reducing the myocardin-dependent promoter activities of SMC marker genes (SM22alpha and caldesmon) [32]. It appears that ALK6 and BMPR2 together mediate MSX1 induction through SMAD1/5/8 activation [21,52]. It is also possible that this might not only be related to BMP receptors, but also to their interaction with integrins [52]. This suggests that in PAH, BMP pathway can potentially upregulate MSX1 expression in pulmonary vascular cells, but the exact mechanism of action needs further investigation.

In conclusion, we find that, in PAH, upregulation of MSX1 leads to pulmonary vessel dropout or dedifferentiation by influencing multiple pathway genes associated with vascular development, in a cell-specific manner. Further understanding the downstream signaling pathways that lead to MSX1 upregulation and understanding molecular consequences of MSX1 upregulation will help to develop effective treatments against PAH disease.

## Data Availability

RNA seq datasets are available at Gene Expression Omnibus, https://www.ncbi.nlm.nih.gov/geo/.
